# γδ T cells take the stage

**DOI:** 10.1002/cti2.1085

**Published:** 2019-10-31

**Authors:** Emily M Eriksson, Martin S Davey

**Affiliations:** ^1^ Division of Population Health and Immunity The Walter and Eliza Hall Institute of Medical Research Melbourne VIC Australia; ^2^ Department of Medical Biology The University of Melbourne Melbourne VIC Australia; ^3^ Infection and Immunity Program and Department of Biochemistry and Molecular Biology Biomedicine Discovery Institute Monash University Melbourne VIC Australia; ^4^ Australian Research Council Centre of Excellence in Advanced Molecular Imaging Monash University Melbourne VIC Australia

## Abstract

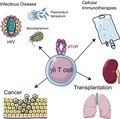

Despite the discovery of the γδ T‐cell receptor (TCR) over 30 years ago, the exact role of γδ T cells in infectious disease, cancer and transplant immunology remains unclear. Nevertheless, γδ T cells are frequently implicated in both anti‐microbial and anti‐tumor immunity.^1^ γδ T cells are formed of innate‐like and adaptive populations, that recognise target cells in a major histocompatibility complex (MHC)‐independent fashion, consistent with a lack of surface CD4/CD8αβ co‐receptor expression. Studies in mice have highlighted ‘innate‐like’ γδ T cells subsets emerging early in thymic development, bearing semi‐invariant TCRs,^1^, ^2^ suggestive of a limited range of self‐ligands. Recent studies in humans have also identified that a major human γδ T‐cell population, constituting the largest subset of γδ T cells at tissue locations, follow an adaptive immunobiology.^3^, ^4^, ^5^ In this Special Feature of *Clinical & Translational Immunology*, we have invited experts in γδ T‐cell biology to provide an overview of the emerging roles of this often overlooked and unique population of T cells in health and disease (Figure 1).

**Figure 1:**
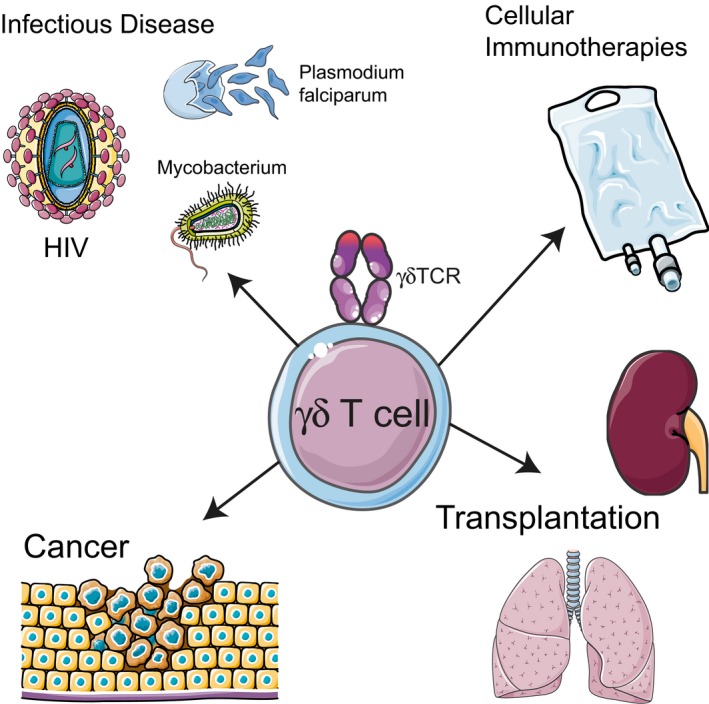
Unconventional immunity provided by γδ T cells. Scheme depicting the multifaceted interactions of γδ T cells in infectious disease, cancer and transplantation. Artwork created from modified material supplied by Servier Medical Art, under a Creative Commons Attribution 3.0 Unported License.

Siegers *et al.* 6 provide a memorial article in commemoration of the late Professor Paul Fisch (1959–2018). Paul was a pioneer of γδ T‐cell research where he made a profound contribution to our understanding of γδ T cells. Paul was one of the first to describe the unique responses of γδ T cells in infection and cancer, and provided evidence suggesting γδ T‐cell recognition events were independent of MHC molecules.

Dantzler *et al.* 7 provide an overview of γδ T‐cell responses in infectious diseases of global health importance, such as tuberculosis, malaria and influenza. This review highlights several recent studies investigating γδ T‐cell responses to vaccines targeting these infections.

Juno *et al.* 8 highlight the impact of acute, chronic untreated and treated HIV infection on peripheral γδ T‐cell subsets and discuss new insight into the potential for harnessing γδ T cells as components of an anti‐HIV immunotherapy.

Raverdeau *et al.* 9 highlight the exciting new avenues for harnessing γδ T cells in anti‐cancer immunotherapies but also underscore evidence for the pro‐tumor properties of γδ T cells.

Sullivan *et al.* 10 review the evidence for γδ T cells in solid organ and haematopoietic stem cell transplantation. The authors focus on their potential roles in allograft acceptance and rejection, as well as their impact on transplant‐associated infection and post‐transplant malignancy.

Together, this collection of reviews highlights current paradigms in γδ T‐cell biology in health and disease. Each article then places γδ T cells in scenarios of infection and immunity – albeit ‘good, the bad and sometimes confusing’ – further emphasising the critical importance of developing a better understanding of this unconventional T‐cell population.

## Conflict of Interest

The authors declare no conflict of interest.
